# Functional analysis of hepatitis B virus pre-s deletion variants associated with hepatocellular carcinoma

**DOI:** 10.1186/1423-0127-19-17

**Published:** 2012-02-07

**Authors:** Chih-Ming Lin, Gen-Ming Wang, Guey-Mei Jow, Bing-Fang Chen

**Affiliations:** 1 School of Medicine, Fu Jen Catholic University, New Taipei City, Taiwan; 2 Department of Surgery, Cathay General Hospital, Taipei, Taiwan; 3 Division of Gastroenterology, Department of Internal Medicine, Shin Kong Wu Ho-Su Memorial Hospital, Taipei, Taiwan

**Keywords:** HBV, hepatocellular carcinoma, pre-S deletion, S promoter

## Abstract

**Background:**

Naturally occurring pre-S deletion mutants have been identified in hepatitis B carriers and shown to be associated with the development of hepatocellular carcinoma. The phenotypes of these pre-S deletion genomes remain unclear, and they were investigated in this study.

**Methods:**

The pre-S deletion genomes: (1) pre-S1 deletion, (2) deletion spanning pre-S1 and pre-S2, (3) pre-S2 N-terminal deletion, and (4) pre-S2 internal deletion were constructed and analyzed by transfection into Huh-7 cells.

**Results:**

Functional analyses reveal that these mutants were divided into two groups: S promoter deletion and non-S promoter deletion variants. Compared with the wild-type genome, S promoter deletion variants led to an inverse ratio of pre-S1 mRNA and pre-S2/S mRNA, and intracellular accumulation of surface proteins. An interesting finding is that a small amount of L proteins was detected in the medium from S promoter deletion variant-transfected cells. Non-S promoter deletion variants conversely displayed a wild-type like mRNA and protein pattern. The secretion of surface proteins from non-S promoter deletion variants was inhibited less than from S promoter deletion variant. Immunofluorescence analysis showed mutant surface proteins colocalized with ER and exhibited an atypical distribution: granular staining pattern in the S-promoter deletion variants and perinuclear staining pattern in the non-S promoter deletion variants.

**Conclusion:**

This study shows that these pre-S deletion genomes exhibit two different phenotypes in mRNA transcription, surface protein expression and secretion. This diversity seems to result from the deletion of S promoter rather than result from the deletion of pre-S1 or pre-S2.

## Background

Hepatitis B virus (HBV) is a small, enveloped DNA virus that causes acute and chronic liver diseases. The majority of acute HBV infections are usually self-limited, whereas patients with chronic HBV infection usually pursue a life-long course. The clinical consequences of chronic HBV infection include chronic carrier state, chronic hepatitis, cirrhosis, and hepatocellular carcinoma (HCC) [1,2]. Host, viral factors and their interactions contribute to the progression of liver disease.

**Figure 1:**
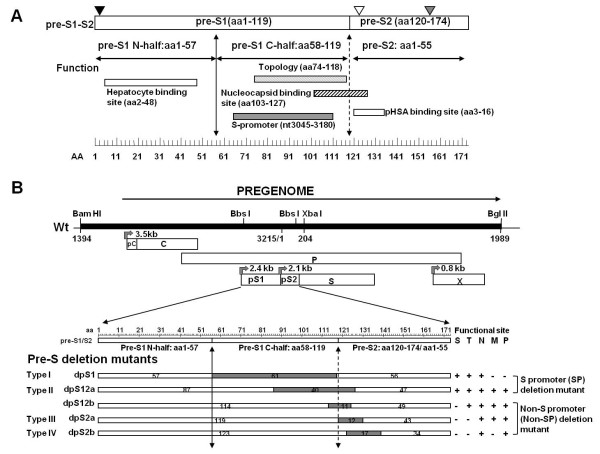
**Map of HBV pre-S region and viral genomes**. (A) Functional domains within the HBV pre-S region. The pre-S region consists of the pre-S1 and pre-S2 domains. The pre-S1 domain contains 119 amino acids (used in this study) and is further divided into two parts, N half (aa 1 to 57) and C half (aa 58 to 119). The pre-S2 domain contains 55 amino acids. The pre-S domain contains multiple functions as shown. N-half of pre-S1 contains hepatocyte binding site essential for infection. C-half of pre-S1 contains a site important for dual topology of L proteins and a nucleocapsid binding site for virion morphogenesis. C-half of pre-S1 also contains S-promoter necessary for expression of S gene. Pre-S2 domain has pHSA (polymerized human serum albumin) binging site. Black triangle, myristylation at second amino acid; white triangle, N-link glycosylation at N-4 of the M protein; gray triangle, O-link glycosylation at T-37 of the M protein. (B) Plasmids encoding the wild-type (wild-type1.2) and pre-S deletion HBV genome. HBV sequence is shown by heavy line and flanking plasmid pGEM-4z sequence shown by the thin line. ORFs for pre-C (pC), C, P, pre-S1 (pS1), pre-S2 (pS2), S, and × genes are drawn as boxes. Arrows above the ORF boxes show the start sites for the pregenomic/Core (3.5 kb), pre-S1 (2.4 kb), pre-S2/S (2.1 kb), and × (0.8 kb) mRNA. Relevant endonuclease restriction sites and positions are indicated. Map of wild-type pre-S1/S2 domain is shown and the number above the map indicates the amino acid site of the defined domain. Gray box indicates the deleted region and the number in the box indicates the length of this box. The deletion of five functional sites are indicated and shown at right hand column. S, T, N, M, and P indicate 5 functional sites, S-promoter, topology, nucleocapsid binding site, the start codon of M protein, and pHSA site respectively. + and - indicate presence of deletion and absence of deletion respectively.

HBV has four open reading frames that encode × protein, DNA polymerase, core, and surface protein. The viral surface proteins compose of three different, yet structurally related surface proteins from a single open reading frame, named large (L), middle (M), and small (S) protein. The S protein is 226 amino acids (aa) in length, and the M and L protein are assembled by amino-terminal extension of 55 aa of the pre-S2 domain and of 163 to 174 aa (depending on the strain) of the pre-S (pre-S1 and pe-S2) domain, respectively [3]. The functions of the pre-S region have been studied previously and summarized in Figure 1A. The pre-S domain of L surface protein plays vital roles in the viral life cycle by e-pre-S (external in secreted envelope) to mediate the attachment of HBV to liver cells, by i-pre-S (internal in the secreted envelope) to perform a matrix-like function in nucleocapsid envelopment, and by exerting various regulatory functions [4-13]. Conversely, the M protein has been demonstrated to be functionally nonessential for viral assembly or DNA replication. The pre-S2 domain of M protein could bind to polymerized human serum albumin (pHSA) (aa 3-16), but the significance of this binding is unknown [13].

Due to the spontaneous error rate of viral polymerase, HBV genome evolves during the course of infection under the antiviral pressure of the host immunity or specific therapy [14]. Previously, it was reported that patients with progressive liver diseases including HCC had a higher frequency of pre-S deletions [15]. Sequencing analysis found the deletions happened more frequently in 3' terminus of pre-S1 and 5'-terminus of pre-S2 regions. Functional mapping of these pre-S deletion sequences showed most of deletion mutants lost one or more of functional sites (shown in Figure 1A), such as pHSA site, nucleocapsid binding site, site for dual topology, S-promoter, and the start codon of M protein. Until now, only a few naturally occurring HBV pre-S1 or pre-S2 internal deletion mutants were cloned and characterized functionally in full-length genome [16-20]. Studies of pre-S1 deletion genomes showed a decrease of pre-S2/S transcripts and an inverse increase of pre-S1 transcripts [16-18]. The impacts of pre-S1 deletion on virion secretion are controversial; two groups showed release virion particles [16,17], but not the other group [18]. Conversely, pre-S2 internal deletion mutants have no effect in RNA transcription, DNA replication, or virion secretion [19]. Furthermore, both pre-S1 and pre-S2 internal deletion mutants have been reported to cause the accumulation of large surface protein in the endoplasmic reticulum (ER), and lead to ER stress [19,21,22]. It was suggested that large amounts of reactive oxygen species are generated through ER stress, and they cause oxidative DNA damage, induce mutagenesis in the genome, and ultimately result in HCC [23].

So far, the functional analysis were restricted in pre-S1 deletion and pre-S2 internal deletion, but the extensive functional studies of full-length genomes with N terminal pre-S2 deletion or pre-S deletion spanning pre-S1 and pre-S2 are absent. In this study, four different types of pre-S deletion mutants (Figure 1B) associated with development of hepatocellular carcinoma: (1) pre-S1 deletion (dpS1), (2) deletion spanning pre-S1 and pre-S2 (dpS12a and dpS12b), (3) pre-S2 N-terminal deletion (dpS2a), and (4) pre-S2 internal deletion (dpS2b) were selected to investigate their impacts on HBV RNA transcription, viral protein expression, surface protein secretion, and intracellular location of surface proteins.

## Methods

### Pre-S deletion subgenomes

The pre-S deletion subgenomes (genotype Ba) derived from five sera of patients with HCC infected with HBV were investigated [15]. The pre-S deletion subgenomes were amplified, cloned into T&A vector (Real Biotech Co., Taipei County, Taiwan), and sequenced as described previously [15,24]. These mutants were divided into four types based on the region deleted (Figure 1B). Type I, the dpS1 mutant has a deletion in the C terminus of pre-S1 region (61 amino acids, aa 58-118). Type II, the dpS12a and dpS12b have a deletion spanning pre-S1 and pre-S2 region. Type III (dpS2a) deletes the N-terminal 12 amino acids of pre-S2. Type IV (dpS2b) is a pre-S2 internal deletion. Based on functional mapping of sequences, two (dpS1 and dpS12a) losing the S promoter were classified as S promoter (SP) deletion variant; the other three (dpS12b, dpS2a, and dpS2b) still having S promoter were classified as non-S promoter (Non-SP) deletion variant. In addition, three (dpS12a, dpS12b, and dpS2a) deleted the start codon of M surface protein.

### Cloning of wild-type and pre-S deletion genomes

Plasmid of wild-type HBV replication-competent genome containing a 1.2-mer HBV genome unit lengths was constructed from a wild-type genotype Ba plasmid (B8w, a generous gift from Dr. Chun-Jen Liu, containing full length HBV genome). Briefly, wild-type genome was constructed by tri-mer ligation. *BamH*I-*Xba*I (nt 1394-204) and *Xba*I-*Bgl*II (nt 204-1989) HBV fragments were cloned into *BamH*I-*Pst*I double digested pGEM-4z vector. Five different pre-S deletion replication-competent HBV genomes were made by insertion of the Bbs I fragment with a pre-S deletion into the wild-type genome following digestion with the BbsI restriction enzymes, respectively. All of restriction enzymes were provided from New England Biolabs.

### Cell culture and transfection

Huh-7 cells were grown at 37°C under 5% CO_2 _in Dulbecco's modified Eagle medium, supplemented with 10% FCS (Gibco Invitrogen Corporation, USA) and 2% L-glutamine. The cells were plated at a density of 1 × 10^6 ^cells per 60-mm dish or 5 × 10^5 ^cells per well in 6-well plates 24 hours prior to transfection. Transfection of cells was performed with lipofectamine 2000 (Invitrogen Life Technologies, CA) following the user guidelines. Cotransfections with enhanced green fluorescent protein (EGFP) reporter plasmid were performed, and the amount of EGFP synthesized was quantified using fluorometer to normalize the transfection efficiency. Cells were harvested at day 3 post-transfection. Calculation of transfection efficiency was performed as follows: equivalent number of viable cells was taken and lysed, and then intracellular EGFP fluorescence was measured. The EGFP in cell culture medium was also measured to examine the possibility of cell lysis. The relative volume of medium from equivalent number of live cells was taken and used to determine the EGFP fluorescence. The presence of cell lysis was calculated by extracellular fluorescence divided by intracellular fluorescence.

### Western-blot analysis of intracellular HBV surface and core proteins

Proteins of transfected cells were harvested, separated in 12% polyacrylamide gels and transferred to PVDF membranes. The membrane was incubated with the primary antibody, and then was incubated with the second antibody, which was conjugated with horseradish peroxidase. The proteins were detected by using ECL chemiluminescence kit (Perkin-Elmer Life Science, Boston, MA). Loading of equal protein amounts was controlled with an anti-actin antibody (Millipore, USA). The primary antibodies used for Western-blot were as follows: anti-HBs; anti-pre-S1, MA18/7, a generous gift from Dr. Gerlich; anti-HBV core, kindly provided by Dr. Hui-Lin Wu (Hepatitis Research Center, National Taiwan University Hospital, Taiwan). Since the anti-HBc was raised by recombinant HBV core protein, it could detect HBV core (MW = 21 kDa) and HBeAg (MW = 15-18 kDa). When the culture supernatant was analyzed, the anti-HBc recognizes the HBeAg more efficiently.

### Extracellular secretion of HBV surface and e proteins in culture supernatants

The amounts of HBV surface proteins (HBsAg) and e protein (HBeAg) in culture supernatants were quantified by using the enzyme-linked immunosorbent assay (ELISA) kit (SURASE b-96 and EASE BN-96 kit, General Biologicals CORP., HSIN-CHU, Taiwan). The supernatants were diluted to keep the amount of HBsAg and HBeAg within the linear range of the assay. All transfections and quantitations were repeated independently at least three times.

The surface proteins and HBeAg in the culture supernatant were concentrated by Amicon Ultra-4 centrifugal filter devices (Millipore, USA) and performed the Western-blot analysis as described above.

### Northern blot analysis

Total RNA was extracted from transfected cells with TRIzol reagent (Invitrogen, USA) following the manufacturer's instructions. Five μg RNA were separated by electrophoresis on a 1% agarose gel and transferred to a nylon membrane. The blot was probed with a DIG-labeled full-length DNA HBV probe. The probe was generated with a PCR DIG Probe Synthesis kit (Roche Diagnostics GmbH, Mannheim, Germany). Detection was done using the DIG luminescent detection kit (Roche), and bands were quantitatively analyzed with the Image J software.

### Immunofluorescence studies

The transfected cells were grown on chamber slides, washed two times in phosphate-buffered saline (PBS) at 4°C, and fixed with 4% paraformaldehyde. After a blocking reaction with 3% BSA, cells were incubated with primary antibodies against pre-S1, S and endoplasmic reticulum (ER) membrane: anti-Calnexin antibody (Cell Signaling technology, Inc., USA) prior to incubation with secondary antibodies (Alexa Fluor 488-labeled anti-mouse IgG and Alexa Fluor 594-labeled anti-rabbit IgG). The nuclei of the cells were counterstained with 1 μg/ml of Hoechst 33258 (Sigma, St. Louis, MO). Images were examined under an Olympus fluorescent microscope.

## Results

### Viral mRNA levels

**Figure 2:**
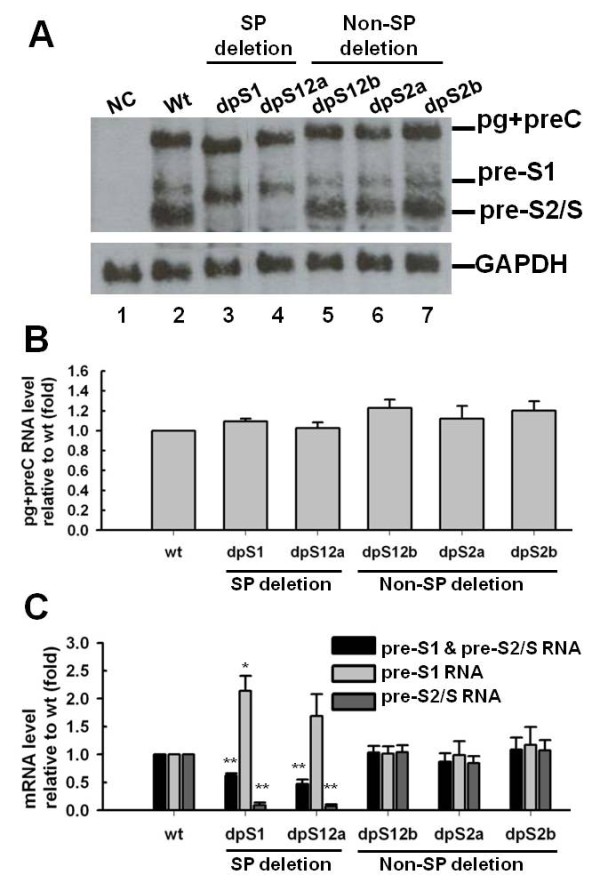
**Analysis of HBV RNA transcripts**. HBV RNA was analyzed three days after transfection of Huh7 cells with wild-type genome or variant HBV DNAs. NC, negative control, only pGEM-4z transfected. (*A*) Exemplary Northern blot of total RNA detected with a full-length HBV DNA probe. GAPDH RNA was detected as loading control. (*B*) Quantitative evaluation of the 3.5-kb pg (pregenomic)/preC (precore)-RNA signals in Northern blots relative to wild-type was analyzed by Image J software. Mean values and standard errors of at least three independent experiments are indicated. (C) Quantitative evaluation of preS1-mRNA and pre-S2/S-mRNA signals relative to wild-type was performed by Image J software. Mean values and standard errors of at least three independent experiments are shown. * indicates *p *< 0.05; ** indicates *p *< 0.01.

To determine the effect of pre-S deletions on HBV transcripts, the HBV RNA levels were analyzed by Northern-blot hybridization of total cellular RNA. A representative blot is shown in Figure 2; all five variants showed similar levels of 3.5 kb pg/preC RNA compared with the control wild-type genome.

As expected, in S promoter (SP) deletion variants (dpS1 and dpS12a), the levels of pre-S2/S mRNA were decreased and the amounts of pre-S1 mRNA were increased compared with the wild-type genome. Non-S promoter (Non-SP) deletion mutants (dpS12b, dpS2a, and dpS2b) conversely showed almost equal levels of total pre-S1 and pre-S2/S mRNAs, pre-S1 mRNA, and pre-S2/S mRNA when compared with the wild-type genome.

### Viral protein expression: intracellular retention of surface proteins

**Figure 3:**
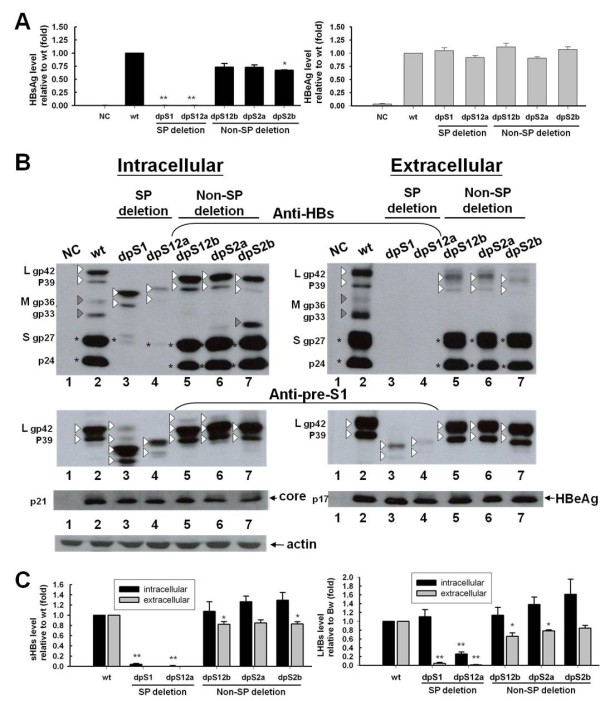
**Analysis of secreted and intracellular HBV proteins three days after transfection of Huh7 cells with wild-type and pre-S deletion variant DNAs**. NC, negative control, only vector pGEM-4z transfected. (A) HBsAg and HBeAg levels in cell culture supernatant (diluted 1:10) relative to wild-type levels detected by ELISA. Mean values and standard errors of at least four experiments are shown. * indicates *p *< 0.05; ** indicates *p *< 0.01. (B) Western blots of cell lysate and cell culture supernatant were used to detect HBV surface proteins with primary antibodies anti-pre-S1 and anti-HBs; HBV core and HBeAg were detected by rabbit polyclonal antibody anti-HBV core. Detection was carried out with peroxidase-coupled secondary antibodies and chemiluminescence substrate. Detection with anti-actin antibody was performed as loading control. The white triangles, gray triangles and asterisks indicate large, middle and small surface protein, respectively. (C) Quantitative evaluation of intracellular and extracellular viral proteins relative to wild-type was analyzed by Image J software. Mean values and standard errors of at least three independent experiments are indicated. * indicates *p *< 0.05; ** indicates *p *< 0.01.

Based on the results of ELISA, a reduced secretion of surface proteins (HBsAg) was reproducibly found from pre-S deletion mutants compared with the wild-type virus, especially from SP deletion mutants (Figure 3A). However, the secretion of another HBV antigen, HBeAg, was less affected (Figure 3A). Since ELISA detects all three (L, M, and S) HBV surface proteins, in order to gain a better resolution, the Western blot analysis using specific antibodies to identify each individual surface protein was performed.

Two monoclonal antibodies were used: (1) anti-HBs is S-specific, and it detects all three (L, M, and S) proteins; (2) anti-pre-S1 is specific for the L protein; the blot was first detected with anti-HBs, stripped, and reprobed with anti-preS1. As shown in Figure 3B, compared with wild-type genome, the SP deletion mutants showed a significantly decreased amount of S protein but similar (dpS1) or lower (dpS12a) level of intracellular L protein (Figure 3B-C). In addition, the secretions of L proteins were remarkably low. Conversely, the Non-SP deletion mutants showed slightly higher levels of intracellular S and L proteins and a slightly decreased amount of extracellular S proteins (Figure 3B-C). Most surprisingly, the extracellular L proteins for Non-SP deletion mutants looked less abundant than wild-type L proteins in the anti-HBs blot, but not in the anti-pre-S1 blot (Figure 3B, anti-HBs and anti-pre-S1 panels). The lack of recognition of mutant L proteins by the anti-HBs was probably due to the alteration of immunogenicity of S domain. Another surprise was that the intracellular mutant L proteins exhibited a more heterogeneous and had additional L proteins with a higher molecular weight than expected, which might be di-, and tri-glycosylated L proteins (Figure 3B, anti-pre-S1 panel). As an internal control, intracellular core proteins or extracellular HBeAg were measured and found to be similar between wild-type HBV and pre-S deletion mutants (Figure 3B, core and HBeAg panels).

### Intracellular localization of HBV surface proteins in pre-S deletion mutant's transfected cells

**Figure 4:**
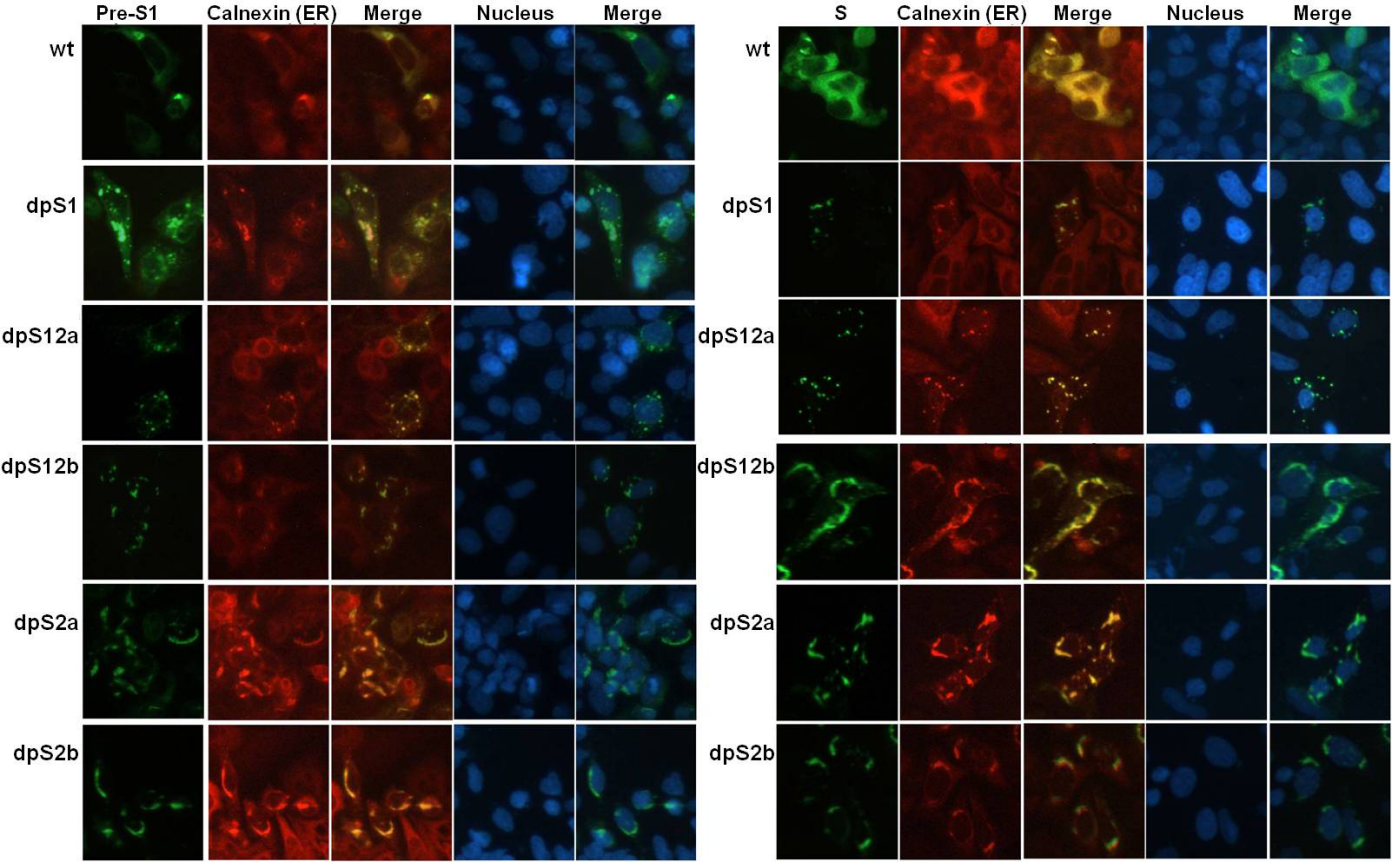
**Immunofluorescence staining of surface proteins in wild-type genome and pre-S deletion mutants -transfected Huh-7 cells**. Huh-7 cells were first seeded on the chamber slide and incubated at 37°C overnight. After transient transfection of wild-type and pre-S deletion mutants, the slides were stained with monoclonal antibodies against pre-S1, anti-pre-S1 and S, anti-HBs. The endoplasmic reticulum (ER) of the cells was double-immunostained with anti-calnexin antibody. The nuclei of the cells were counterstained with Hoechst. Images were examined under an Olympus fluorescent microscope (original magnifications x200).

To investigate the intracellular location of the surface proteins, double-immunofluorescence staining with cellular proteins of ER compartment was performed (Figure 4). Cells transfected with wild-type constructs showed a diffuse and homogenous staining pattern of surface proteins. In contrast, a granular staining of surface proteins was observed for SP deletion variant-transfected cells (dpS1 and dpS12a). The results for Non-SP deletion variants (dpS12b, dpS2a, and dpS2b) were different from those of wild-type and SP deletion constructs. The distribution of surface proteins showed a perinuclear staining pattern. The staining patterns of calnexin (ER) seemed to be changed by transfecting with different pre-S deletion mutants. It is not surprising since previous study showed that the L proteins colocalized with and bind to calnexin [25], the distribution of calnexin would be changed by mutant L proteins.

## Discussion

Recently many studies demonstrated the association of pre-S deletion with the development of progressive liver disease [15,22,26-28]; however, most of molecular characteristics of these pre-S deletion genomes remain unknown. In this study, we analyzed the phenotype of five naturally occurring pre-S deletion mutants: one (dpS1) is pre-S1 deletion mutant; two (dpS12a and dpS12b) delete the border between pre-S1 and pre-S2; one is pre-S2 N terminal deletion mutant (dpS2a); one is pre-S2 internal deletion mutant (dpS2b). Based on functional mapping of pre-S sequences, they are also divided into two groups: two (dpS1 and dpS12a) are S promoter (SP) deletion variants, and three (dpS12b, dpS2a, and dpS2b) are non-S promoter (Non-SP) deletion variants. The Northern blot experiments showed that all pre-S deletion genomes produced comparable amounts of pregenomic RNA as found for the wt HBV construct. However, an altered transcription with reduced levels of pre-S2/S mRNA was found in S promoter deletion variants. It is known that the surface gene of HBV is controlled by 2 different promoters: the pre-S1 and the S promoter, which regulate the transcription of 2.4-kb pre-S1 mRNA and 2.1-kb pre-S2/S mRNA, respectively. The pre-S1 promoter is located 5' of the first in-frame ATG of the L surface protein and regulated by at least two liver-enriched transcription factors, HNF-1 and HNF-3. The S promoter is a TATA-less promoter and found within the coding region of the pre-S1 region. It is reported that a CCAAT element in the S promoter and its 3' terminus are essential for high-level expression of 2.1-kb pre-S2/S mRNA [29]. Furthermore, previous study demonstrated that this CCAAT element could not only increase the amount of pre-S2/S transcripts, but also decrease the amount of pre-S1 transcripts [30]. Therefore, deletion of this element would decrease the amount of pre-S2/S transcript, increase the amount of pre-S1 transcript, and cause an inverse ratio of pre-S1 mRNA to pre-S2/S mRNA. Indeed, two S promoter deletion variants (dpS1 and dpS12a) caused a decrease of pre-S2/S transcripts and an inverse ratio of pre-S1 mRNA to pre-S2/S mRNA, as expected. Conversely, when non-S promoter deletion variants (dpS12b, dpS2a, and dpS2b) were transfected, the ratio of pre-S1 mRNA to pre-S2/S mRNA was similar between wt genome and variants.

Synthesis and secretion of surface proteins also showed two different patterns. The phenotype of the non-S promoter deletion mutants (dpS12b, dpS2a, and dpS2b) did not appear to differ significantly from wild-type with respect to intracellular and extracellular abundance of S and L protein (Figure 3A and 3B, lane 5-7 vs. lane 2). Conversely, in S promoter deletion variant-transfected cells, Western-blot analysis showed that the synthesis of S proteins was relatively lower, the M proteins were undetectable, and intracellular accumulation of mutant L proteins were serious (Figure 3B, lane 3-4 vs. lane 2 and Figure 3C). This phenomenon is expected and similar to previous studies [16-18,21]. Intracellular accumulation of L surface proteins results from the fact that the L surface protein cannot be secreted by itself. It needs to complex with S and M proteins to form subviral particles or mature virions for secretion [31]. The behavior of L proteins depends on the relative amounts of the various surface proteins: a small relative amount of L surface protein results in secretion, while a large amount results in retention. Therefore, low level of S protein and absence of M protein would lead to intracellular retention of mutant L proteins.

It is surprising that a small amount of mutant L proteins could be detected in the medium from S promoter deletion variant-transfected cells by Western blotting using anti-pre-S1 antibody. The simplest explanation is that the extracellular mutant L proteins were due to cell lysis rather than secretion of enveloped viral particles. To examine this possibility, cells were cotransfected with a plasmid that expresses a nonsecretable EGFP (enhanced green fluorescent protein) protein, and then the amount of EGFP released from cells was quantified by a fluorometer. Very little EGFP was released into the medium from S promoter deletion variant-transfected cells (less than 0.05% of the amount within the cells), and the amount was identical to the amount found in the medium from wild-type HBV and vector-transfected cells. These results indicated that the extracellular L proteins would not come from cell lysis. Furthermore, similar mutants with S promoter deletion were shown to secrete viral particles by immunoprecipitation and Southern blotting analysis of the culture supernatants [16,17]. Therefore, the extracellular L proteins may be derived from extracellular mature virion. Another surprise is that the S promoter deletion mutants (dpS1 and dpS12a) showed increased pre-S1 mRNA (Figure 2A and 2C), but no increase in intracellular mutant L proteins in Figure 3B (lane 3-4 vs. lane 2). The reason for this difference is probably due to the posttranscriptional regulation. One possible explanation for the decrease in mutant L proteins is that these mutant proteins were transported to proteasomal degradation. Simsek et al. showed that the failure to process N-glycan caused the glycosylated and unglycosylated L and M surface proteins to aggregate and get degraded by proteasomal degradation pathways [32]. Indeed, Western-blot analysis showed that intracellular mutant L proteins exhibited a more heterogeneous and had additional L proteins with a higher molecular weight than expected, which might be di-, and tri-glycosylated L proteins (Figure 3B, anti-pre-S1 panel). The reduction of intracellular mutant L proteins might be resulted from the proteasomal degradation of misfolded unglycosylated and glycosylated L proteins.

Immunofluorescence analysis showed that mutant surface proteins colocalized with the ER and revealed an atypical distribution: a granular staining in the S-promoter deletion variants and a perinuclear staining pattern in the non-S promoter deletion variants. Previously, Wang et al. identified two types of ground glass hepatocytes (GGHs) and found that type I GGHs consistently harbored mutants with deletion over the pre-S1 region (Δ1), whereas type II GGHs contained mutants with deletions over the pre-S2 region (Δ2) [33]. They cloned these pre-S subgenomes and found that these deletion mutants accumulated the surface proteins in endoplasmic reticulum (ER) resulting in strong ER stress. They suggested that large amounts of reactive oxygen species are generated through ER stress, and they cause oxidative DNA damage, induce mutagenesis in genome, and ultimately result in HCC [23]. The morphology of the S promoter and non-S promoter deletion variants is similar to that observed in Δ1 and Δ2 mutants respectively. To examine whether ER stress was induced in our pre-S deletion mutant-transfected cells, the synthesis of ER-stress signal proteins (grp78, IRE1α, and grp94) was analyzed by Western-blot analysis. The results showed that wt HBV genome and pre-S deletion mutants had an enhanced expression level of ER stress signal proteins compared with negative control-vector transfected cells (data not shown). Therefore, these HCC-associated pre-S deletion mutants might behave like the Δ1 and Δ2 subgenomes, causing the mutated surface proteins accumulated in ER, resulting in the ground glass morphology and inducing physiological stress, which may lead to cancer formation finally.

The biological significance of pre-S deletion mutants been explored and suggested that different type of pre-S deletion mutants may exhibit differential biological activities [22]. Su et al. proposed that pre-S deletion mutants can induce ER stress signals, which may result in oxidative stress and DNA damage, and lead to genomic instability. These pre-S deletion mutants may also activate two signal pathways to prevent the hepatocytes from apoptosis, one involving NF-κB to upregulate cyclooxygenase-2 (COX-2) and the other using VEGF to activate Akt/mammalian target of Rapamycin (mTOR) signaling [34]. Pre-S2 internal deletion mutant can additionally induce an ER stress-independent c-Jun activation domain binding protein 1 (JAB1)/p27/retinoblastoma (Rb)/adenovirus E2 promoter binding factor/cyclin A signal to initiate cell cycle progression. Besides, pre-S deletion often occurred in B-cell and HLA-restricted cytotoxic T cell epitopes. These deletions might cause alteration of immune target sites, lead to escape from immune surveillance or reduced binding affinity by MHC I-mediated presentation of modified oligopeptides on the cell surface of hepatocytes, and result in persistent infection.

## Conclusions

This study demonstrates that these pre-S deletion mutants exhibit two altered phenotype, and the diversity seems to result from deletion of the S promoter. Functional analysis of full-length pre-S deletion genomes would not only promote the progress in viral knowledge and pathogenesis, but also help us to battle this enemy. However, definitive answers regarding the role of these mutants in hepatocarcinogenesis await further experiments in the primary hepatocytes or animal models.

## Competing interests

The authors declare that they have no competing interests.

## Authors' contributions

CML and GMW performed most experiments. GMJ provided suggestions and revised the article. BFC contributed to the study concept, research design, data interpretation, and article writing as well as approved the final manuscript. All authors read and approved the final version of the manuscript.
